# Preventing *Candida albicans* Contamination on Packaged Ti-6Al-4V Alloy Surfaces by Cold Atmospheric Plasma Treatment

**DOI:** 10.1021/acsabm.4c01422

**Published:** 2025-02-17

**Authors:** Isau Dantas Morais, Luiz Emanuel Campos Francelino, Vanesca G. S. Leite, Gabriel M. Martins, Jussier de Oliveira Vitoriano, Francisco Marlon C. Feijó, Caio S. Santos, Moacir F. de Oliveira, Clodomiro Alves Júnior, Carlos Eduardo Bezerra de Moura

**Affiliations:** 1 Department of Animal Sciences, Federal Rural University of the Semi-Arid (UFERSA), Mossoró, Rio Grande do Norte 59625-900, Brazil; 2 Department of Health Sciences, Federal University of Rio Grande do Norte (UFRN), Natal, Rio Grande do Norte 9078-970, Brazil; 3 Aeronautics Institute of Technology, São José dos Campos, São Paulo 12228-900, Brazil; 4 Plasma Laboratory Applied to Agriculture, Health and Environment, UFERSA, Mossoró, Rio Grande do Norte 59625-900, Brazil

**Keywords:** nonthermal plasma, biomaterials, biocompatibility, materials testing, titanium, antifungal agent

## Abstract

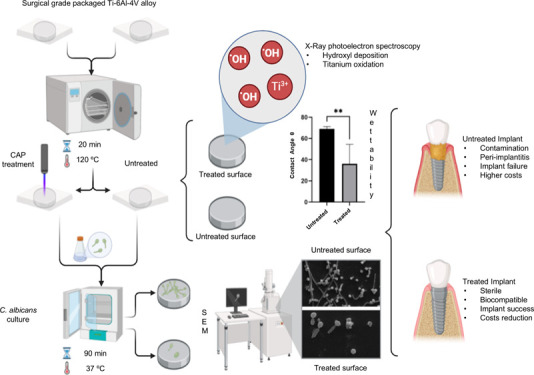

Recent investigations have demonstrated that opportunistic fungi, such as *Candida albicans*, are associated with the contamination of implantable devices, biofilm formation, and consequent resistance to antimicrobial treatment. Preventing biofilm formation on implantable device surfaces represents a significant challenge in medicine and dentistry. This study aimed to evaluate the effects of cold atmospheric plasma (CAP) treatment on Ti-6Al-4V alloy surfaces, sterilized in an autoclave at 120 °C for 20 min in surgical-grade paper packaging, focusing on their potential to optimize surface physicochemical properties and reduce *C. albicans* colonization. X-ray photoelectron spectroscopy (XPS) revealed the formation of Ti–O–H peaks and the oxidation of titanium (Ti^3+^ to Ti^4+^) on CAP-treated surfaces. Sessile drop tests demonstrated a significant improvement in wettability, with a reduction in contact angle (68.94° vs 36.1°, *p* < 0.05). Microbiological assays showed a reduction in *C. albicans* colony-forming units (CFUs) (42,500 ± 8,838 vs 24,000 ± 7,920; *p* < 0.05) and a decrease in pseudohyphae formation (32.7 ± 9.7 vs 11.6 ± 1.8; *p* < 0.05). Scanning electron microscopy (SEM) further confirmed a reduction in yeast aggregates on treated surfaces incubated with fungal strains for 90 min. Data normality was assessed using the Shapiro-Wilk test, and statistical comparisons were performed with *t* tests at a significance level of *p* < 0.05. These findings suggest that CAP is a promising tool for enhancing surface wettability and reducing fungal contamination on Ti-6Al-4V implants sealed in surgical-grade paper, offering potential benefits for medical and dental applications.

## Introduction

Biomaterials are materials used in medicine with wide applications, capable of partially or completely replacing organic tissue, providing benefits to patients, such as increased longevity and improved quality of life.^1^ Titanium and its alloys are noteworthy among biomaterials, due to their high mechanical and corrosion resistance, good malleability, and biocompatibility, which make them widely employed in orthopedics and dentistry.^2,3^

Recent biomaterial research has focused on developing multifunctional surfaces that not only promote efficient colonization by various cell types, such as osteoblasts, fibroblasts, macrophages and endothelial cells, but also inhibit the colonization of infectious agents,^4^ especially in the preoperative and intraoperative periods, thus ensuring implant success.^5^

Implant infections are commonly associated with bacteria present in the clinical environment or in patient tissues.^6^ Fungal implant contamination also represents a significant risk due to long treatment periods and pharmacological resistance following biofilm formation, resulting in persistent and/or recurrent infections, with serious patient health consequences^7,8^*Candida albicans* is noteworthy among fungi responsible for implant and prosthesis contamination, as it is present in mucous membranes, responsible for up to 67% of oral infections in denture users and 60–65% of infections in patients with orthopedic prostheses, with diverse clinical consequences, in addition to being an opportunistic peri-implant lesion agent.^9,10^ Considering the oral mucosa, *C. albicans* is present in 75% of healthy individuals.^11,12^ The main characteristic that contributes to *C. albicans* virulence is its ability to adhere to and form biofilms on biomaterial surfaces when in contact with the mucosa. Following yeast adherence to titanium surfaces, pseudohyphae and hyphae proliferation, biofilm formation and yeast dispersion take place.^8,13^ It is assumed that adhesion is more frequent in titanium implants, due to the chemical composition and hydrophobic properties of these surfaces, since *C. albicans’* surface also has a hydrophobic character, which has a positive correlation with its adhesive capabilities and biofilm formation on materials and cells hydrophobic surface.^5,11,12,14^

Different implant sterilization methods have been employed and explored over the years, including autoclaving, ethylene oxide sterilization, radiation, chemical disinfection, and UV light. While these methods are effective for ensuring sterility during manufacturing, they display significant disadvantages, such as surface oxidation, high cost, chemical residue persistence, prolonged exposure time, and poor surface coverage of complex materials.^15−18^ Furthermore, such methods do not address atmospheric contaminations, such as hydrocarbon deposition, that occur after packaging and reduce the hydrophilicity of titanium surfaces, favoring fungal adhesion^14,19^ To overcome these limitations, research into complementary surface treatments has advanced, with plasma-based methods emerging as particularly promising due to their versatility.^4^

Conventional plasma is generated by applying a voltage between two electrodes in a hermetically sealed system at sufficiently low pressure, resulting in a high-temperature jet. Low-pressure plasma technology has been used as a surface treatment to improve cell adhesion properties and increase surface wettability, improving biocompatibility.^2,6,20^ Furthermore, this method has also been reported as useful in inhibiting and combating bacterial contamination.^21−23^ However, this system requires complex equipment and handling by trained professionals, making it less versatile for treatments outside the laboratory. Furthermore, chemical modifications conducted in a high-energy vacuum environment often result in the disruption of beneficial topographical biomaterial characteristics, in addition to degradation over time, losing treatment effects compared to recently prepared surfaces.^4^

As an alternative, cold atmospheric plasma (CAP) generated by a dielectric barrier discharge (DBD) is emerging. This plasma is formed at low temperatures and does not require a low-pressure chamber, making it portable and enabling its application in outpatient clinics and surgical centers immediately prior to implant application.^4^ Furthermore, because it displays a purely chemical characteristic, it is rich in reactive nitrogen and oxygen species (RONS), which increase microbial titanium implant decontamination efficiency.^24^

Surgical-grade paper packaging, composed of laminated polypropylene and polyester films, plays a fundamental role in maintaining the sterility of packaged products, creating an effective barrier against microorganisms. Thus, treating metal samples inside hermetically sealed packaging can encompass an innovative way to modulate plasma-activated Ti-6Al-4 V implants immediately before their use. This approach was presented by Martins et al. (2024) as promising alternative to increase the effectiveness of the sterilization process and the quality of dentistry implants made of titanium and its alloys, since aging treatment effects are reduced to a minimum.

**Scheme 1:**
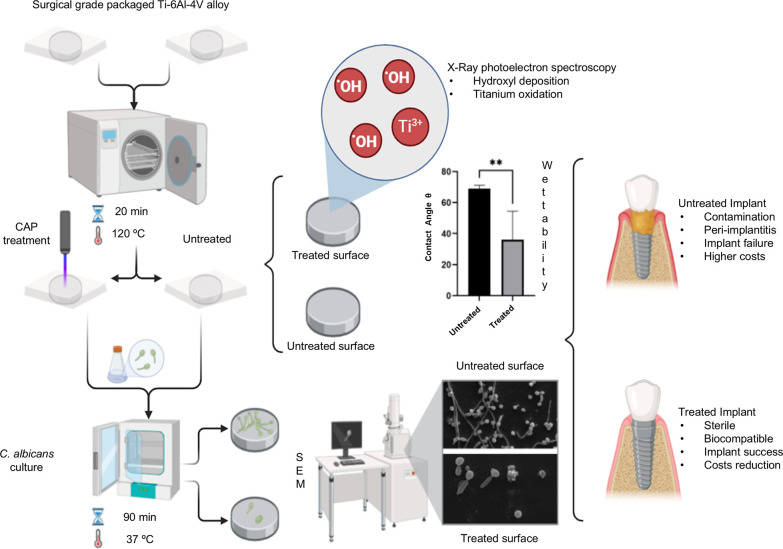
Schematic representation of the experimental workflow for evaluating the effect of cold atmospheric plasma (CAP) treatment on the surface properties of Ti-6Al-4V alloy for preventing C. albicans adhesion.

In this context, this study analyzed the effects of CAP treatment, generated by a portable device, on Ti-6Al-4 V alloy surfaces previously sterilized and wrapped in surgical grade paper (see Scheme 1). The treatment was designed to be applied immediately before clinical use to modulate surface properties, minimize atmospheric contamination within hermetically sealed packaging, and reduce *C. albicans* colonization on titanium surfaces.

## Material and Methods

### Experimental Groups

This study utilized 34 disc-shaped samples of the Ti-6Al-4 V alloy, provided by the national implant company AS Technology (Titanium Fix). All samples were prepared under identical conditions to ensure consistency across analyses. For wettability and XPS assessments, 14 samples were used, divided into a control group (n = 7) and a treatment group (n = 7), with two samples from each group allocated for XPS and five for wettability. Microbiological tests were performed on another 10 samples, equally divided into a control group (n = 5) and a treatment group (n = 5). Morphological evaluations were conducted on the remaining 10 samples, also divided between control (n = 5) and treatment (n = 5) groups, with three fields per sample examined to ensure robust data collection.

### Sample Preparation

Ti-6Al-4 V alloy discs, 9 mm in diameter and 1 mm in thickness, were supplied by Titaniumfix A.S. Technology Componentes Especiais Ltd.a. The discs were gradually sanded with silicon carbide (SiC) sandpaper with different grain sizes (220, 440, 600, 1500, and 2000 MESH), followed by polishing employing a colloidal solution of 40% silica (0.03 μ) and 60% hydrogen peroxide at 30% for 30 min. Subsequently, all samples were cleaned sequentially by immersion in an enzymatic detergent, 70% alcohol and deionized water in an ultrasonic bath for 10 min each. The cleaned samples were then packaged in surgical-grade paper and sterilized in an autoclave at 120 °C for 20 min. Finally, the sterilized samples were stored in a desiccator until CAP treatment.

### Cap Treatment

The surface treatment was carried out by a plasma jet produced by DBD discharge, described in detail by Alves-Junior et al. (2016).^25^ Briefly, the samples wrapped in surgical grade paper were treated with CAP for 15 min. The plasma application distance was 10 mm. The plasma jet was generated by a 13 kV discharge and a frequency of 600 Hz, applied over a flow of 1.5 L/min of high purity helium. To ensure that the treatment was performed by sweeping the entire surface, CAP was applied at five radial points and evenly distributed over the discs for 3 min at each point.

### Sample Characterization

Wettability was assessed using the sessile drop test, performed by observing the angle formed by a 20 μL drop of deionized water pipetted onto the samples by capturing the image with a goniometer video camera, with the contact angle being defined using the ImageJ program.^26^ The analysis was performed immediately after sterilization for the control group and after CAP treatment for the experimental group.

The chemical composition of the polished and CAP-treated Ti-6Al-4 V alloy surfaces were evaluated by XPS using Al Kalpha radiation (1486.6 eV) on an Omicron-SPHERA station. Survey spectra were acquired at a pass energy of 50 eV. High-resolution spectra of specific regions were acquired at a pass energy of 10 eV. The C 1s signal of adventitious carbon at 285 eV was used as an internal energy reference.

### CFUs Cultivation and Counting

The inoculum was prepared using a standard *C. albicans* strain (ATCC 10231), grown aerobically in Brain Heart Infusion broth (BHI, KASVI) at 37 °C in an incubator for 48 h. After incubation, the suspension was adjusted in sterile 0.85% sodium chloride solution until reaching an absorbance of 0.08 to 0.1, equivalent to a concentration of 10^6^/mL (turbidity standard 0.5 on the McFarland scale), confirmed by a spectrophotometer at 530 nm. Aliquots (500 μL) of the *C. albicans* suspension were inoculated onto CAP-treated (n = 10) and untreated (control, n = 10) specimens arranged in a sterile 24-well plate and incubated aerobically for 90 min (adhesion phase) at 37 °C. Subsequently, the suspensions were removed from each well and gently washed twice with sterile saline (0.85%) to remove nonadherent cells. The samples were then sonicated in 9 mL of NaCl solution (0.85%) (dilution 10–1) to detach adherent yeast cells and subjected to serial dilutions to 10^–6^. Using the drop plate technique, 10 μL microdrops from each dilution were inoculated in duplicate onto plates containing Sabouraud Agar supplemented with chloramphenicol, which were then incubated for 48 h at 37 °C. After this, the colony forming units (CFUs) of the 10^–2^ and 10^–3^ dilutions were counted, followed by calculation of the means, and the data were expressed as CFU/mL.

### Morphological Analysis

After inoculation and incubation for 90 min, five treated and five control discs, embedded in a 2.5% glutaraldehyde solution in phosphate buffer (pH 7.4) at room temperature for 12 h for cell fixation, followed by postfixation with osmium tetroxide for 1 h. Subsequently, they were washed with distilled water and dehydrates in an ethanol series at increasing concentrations (25%, 50% and 75%) for 20 min each, followed by immersion in absolute ethanol for 60 min. After dehydration, the samples were metallized with a 9 nm gold film to allow visualization on a scanning electron microscope (Tescan Vega3, TESCAN ORSAY HOLDING, Brno, Czech Republic). Six SEM images were taken per sample, three fields at x1000 magnification and three at x2000 magnification, with pseudohyphae counts performed manually at x2000 magnification by a collaborator and confirmed blindly by two independent researchers.

### Statistical Analysis

The CFU counting, wettability and morphological evaluation assays were performed in quintuplicate. The collected data were evaluated for normality by the Shapiro-Wilk test. After confirming data distribution, the data were submitted to the *t* test at a significance level of *p* < 0.05. All analyses were performed using the Graphpad prism software version 10.1.

## Results

### Wettability

**Figure 1:**
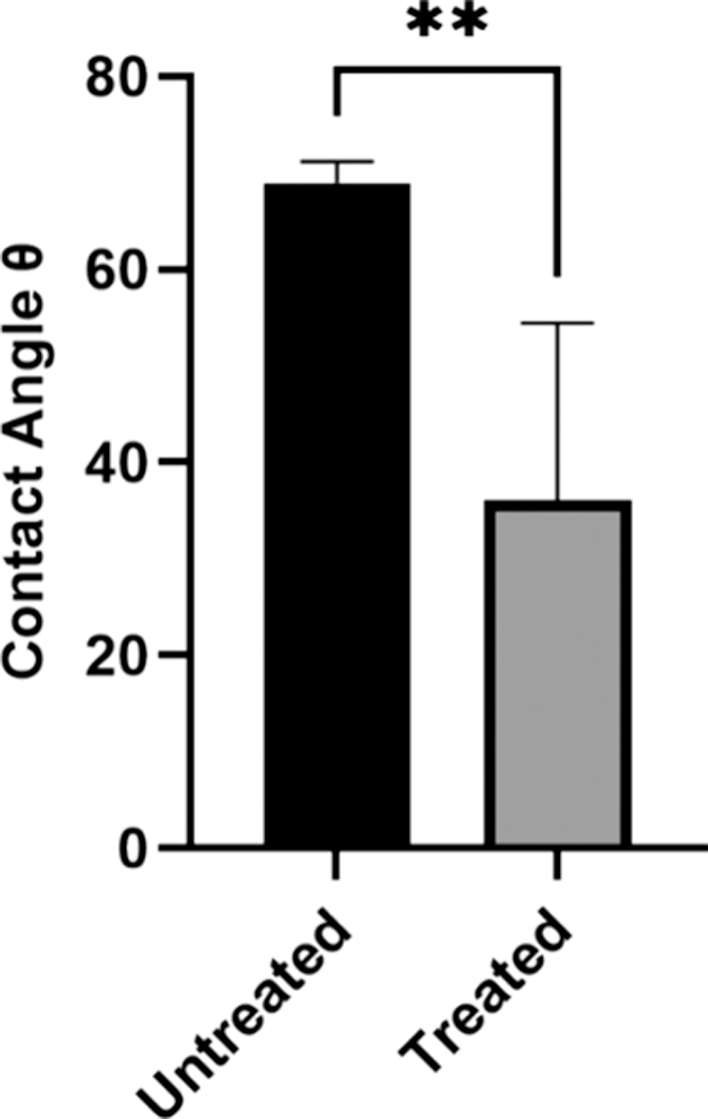
Contact angle of untreated samples compared to CAP-treated samples. **p < 0.001 indicates a significant difference between untreated and CAP-treated samples (*t* test).

The sessile drop test indicated a significant reduction in the contact angle between the deionized water drop on the control surfaces compared to the CAP-treated surfaces (Figure 1).

### XPS Analyses of Ti-6Al-4 V Alloy Samples

**Figure 2:**
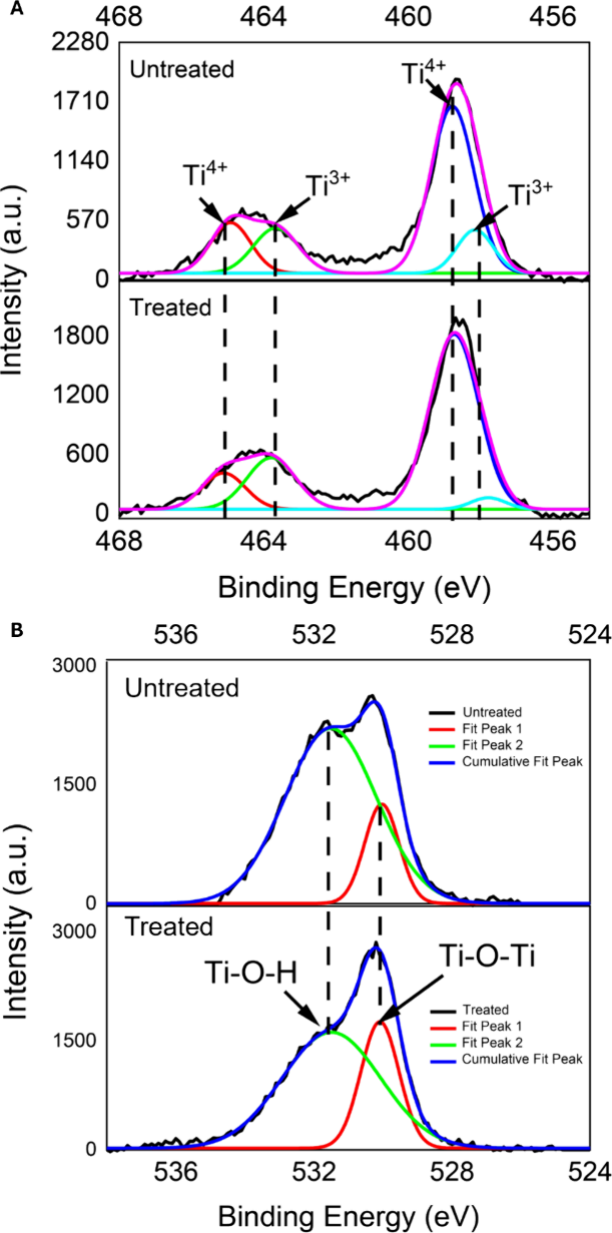
A - XPS analysis of untreated and treated samples, evidencing the effects of CAP in promoting Ti^4+^ to Ti^3+^ oxidation. B - XPS analysis showing the adhesion of −OH- ions to the surface of the treated alloy in relation to the untreated ones.

The XPS analysis indicated that exposure of the Ti-6Al-4 V alloy samples to CAP promoted the oxidation of Ti^3+^ to Ti^4+^ as a result of the action of reactive oxygen species (ROS) formed by the plasma jet. Furthermore, the breaking of Ti–O–Ti bonds was observed, releasing free radicals and forming new functional groups capable of interacting with the hydroxyl groups formed by CAP. This contact resulted in peaks of Ti–O–H groups that remained energetic in the samples that received the CAP intervention (Figure 2).

### UFC *C. albicans* Counts

**Figure 3:**
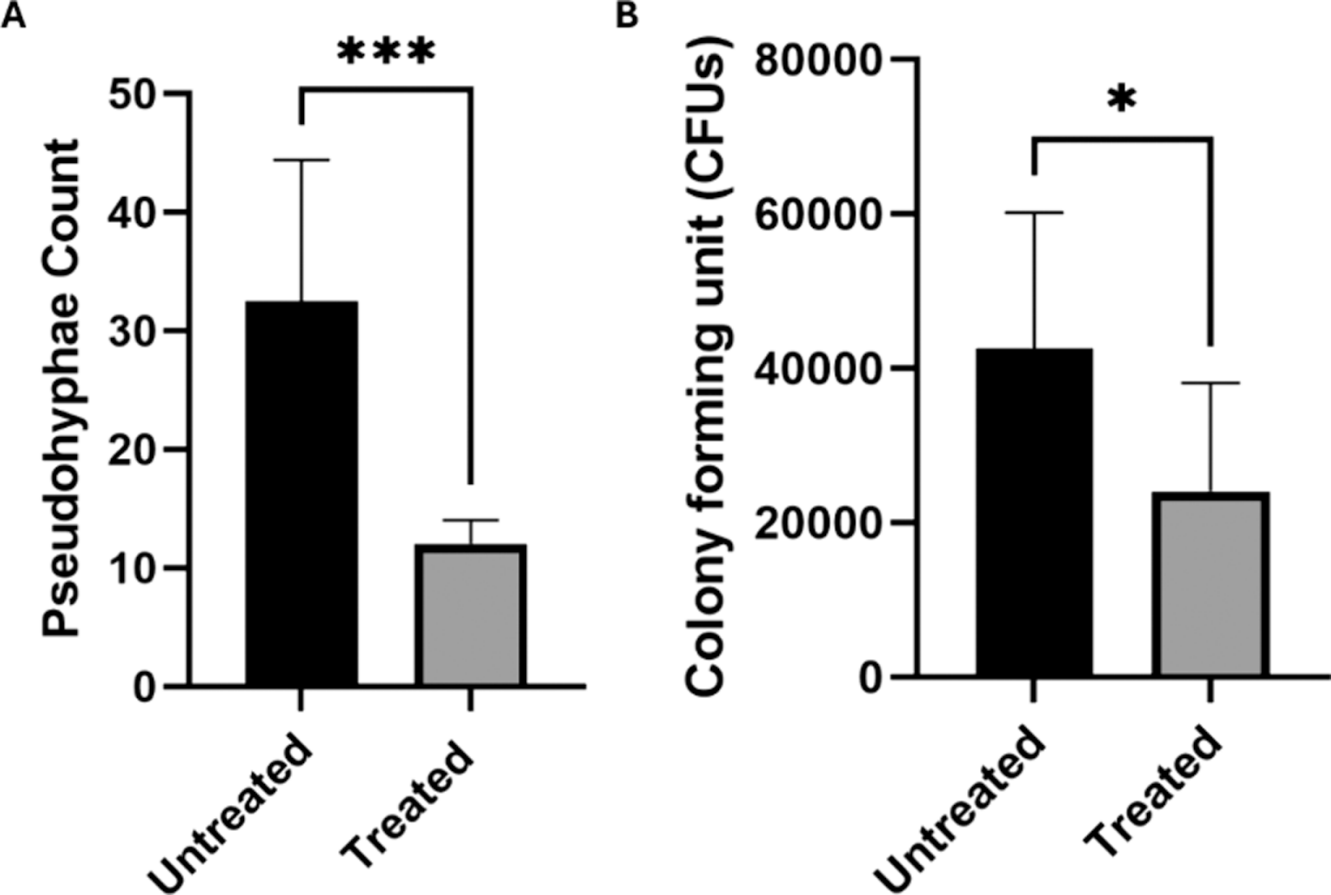
A - mean ± SD CFU counts in untreated samples compared to treated samples. B – mean ± SD pseudohyphae formed after the adhesion phase on the surface of treated and untreated implants. *p < 0.05; ***p < 0.0001 indicates a significant difference between untreated and CAP-treated samples (*t* test).

A reduction in the number of *C. albicans* CFUs was observed on the treated surfaces after 90 min of incubation when assessing the effect of the CAP treatment on the ability of *C. albicans* to adhere to the alloy surface (Figure 3).

### Morphological *C. albicans* Assessment

**Figure 4:**
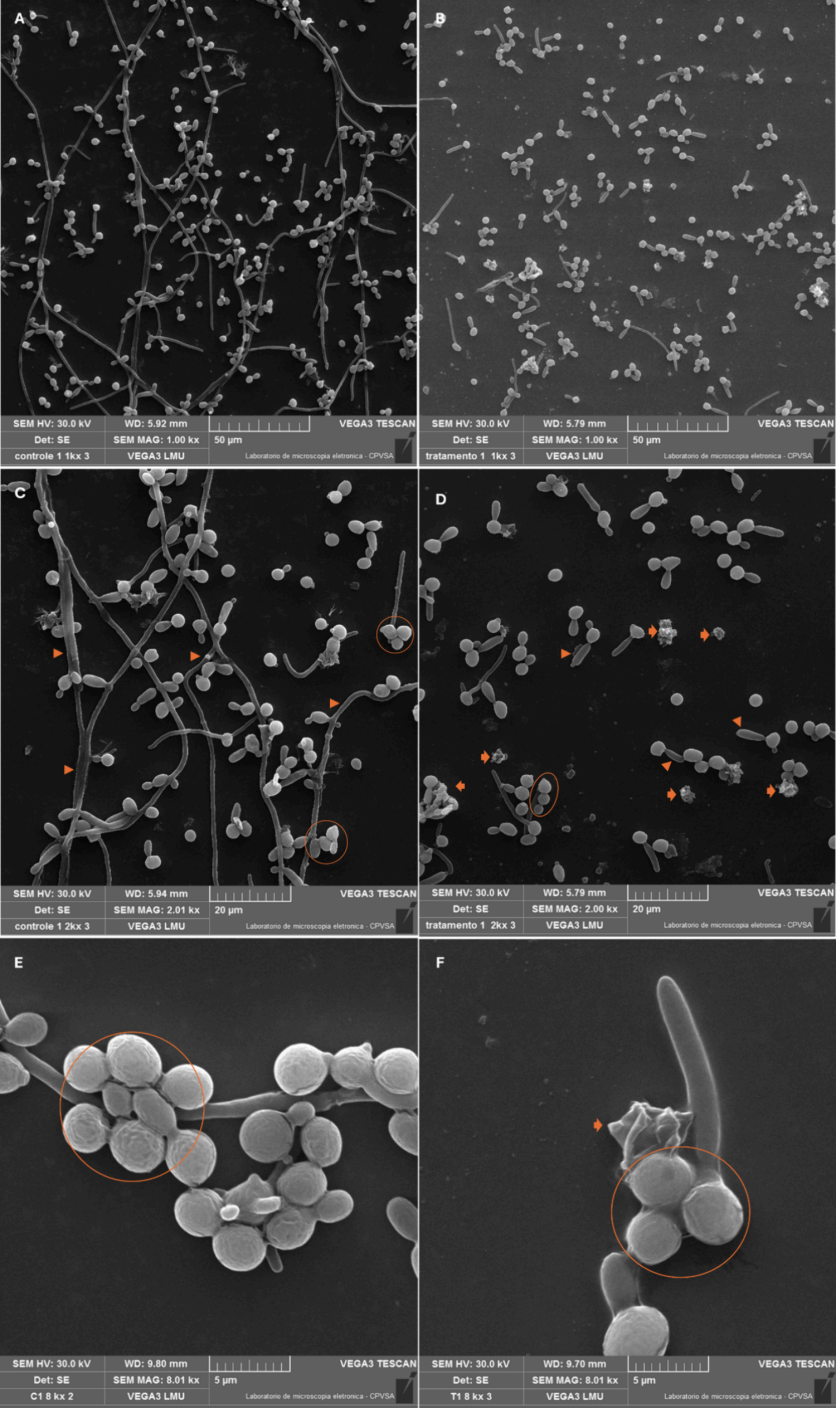
Morphology of *Candida albicans* adhered to Ti-6Al-4 V surfaces. A - untreated sample; B - treated sample; C – zoom in with image details A; D - zoom in with image details B; E - detail of regular yeast aggregates in pseudohyphae on an untreated surface; F - detail of a small aggregate on a yeast-treated surface with an irregular surface, indicating a degenerative process. Circle - yeast aggregate; thick arrow - cell with degenerative appearance; arrowhead - hyphae and pseudohyphae.

The morphological SEM analysis demonstrated a significant reduction in the number of formed pseudohyphae and yeast aggregates adhered to the treated surfaces, as well as irregularities on the surface of some yeast, which acquired a rough appearance, indicative of a degenerative process (Figure 4).

## Discussion

THE CAP treatment of surgical-grade packaged Ti-6Al-4 V implants promoted chemical composition changes of the sample surfaces, resulting in the oxidation of titanium ions and formation of hydroxyl groups observable through XPS. A consequent increase in the wettability of the treated surfaces was noted, leading to decreased *C. albicans* adhesion and aggregation capacity when compared to the control samples.

Wettability refers to the ability of a liquid to wet a solid, with surfaces classified by water contact angle as superhydrophobic (θ>150°), hydrophobic (90° < θ < 150°), hydrophilic (10° < θ < 90°), or superhydrophilic (θ < 10°). This property is commonly associated with the biocompatibility of implant surfaces,^27,28^ important to ensure osseointegration success.^4,28^ All surfaces of the Ti-6Al-4 V alloy samples assessed herein presented a hydrophilic pattern (<90°), although the CAP treatment applied to the surface enhanced this property compared to untreated samples (68.94° vs 36.1°) (Figure 1).

The chemical composition of an implant surface is responsible for its hydrophilic or hydrophobic characteristics and plays a fundamental role in biocompatibility and microbiological adhesion.^5,29^ Increasing surface wettability can reduce or even block the colonization of microorganisms and consequent biofilm formation.^30,31^ The XPS analyses verified that the applied CAP treatment increased the proportion of titanous ions (stable) in relation to titanic ions (nonstable) on the total surface area. Furthermore, the treatment proved effective in modulating the surface by adding hydroxyl ions (−OH^–^) to the elementary structure of the discs. These chemical changes lead to a more hydrophilic surface, as confirmed by the sessile drop test, which, in turn, was capable of rendering the initial *C. albicans* adhesion inefficient in the CAP-treated samples, since yeast depend on hydrophobic surfaces for effective adhesion.^8,9,32^ This was confirmed by the CFU/mL reduction of the treated samples compared to the control (Figure 3).

The SEM results obtained expanded those obtained by CFU counts (Figure 3), with a reduction in the average number adhered to the treated and untreated surfaces (Figure 3) and pseudohyphae proliferation, as well as the formation of yeast-like aggregates on the treated surfaces (Figure 4), all essential factors for the formation of *C. albicans* biofilms on the implant surface.

Biofilm formation is strongly associated with failures in the implantation of metal prostheses, as well as the development of antimicrobial resistance.^7,8,10^ Hyphal formation depends on the events that follow the initial yeast contact with surfaces (which takes place in the first 90 min after the initial contact of the *C. albicans*)^8^ such as attraction and repulsion forces and electrostatic interactions, in addition to the expression of adhesion molecules produced by yeast and hyphal formations for aggregation between yeasts and cell–substrate adhesion, in which the hydrophilic character of the surface being an important variable in reducing the adhesive capacity of *C. albicans.*(7,8) Thus, the treatment of the sample surface within the established parameters proved to be efficient in reducing the adhesion capacity of the applied *C. albicans* strain, when incubated for 90 min on the treated surfaces, as well as the proliferation of its pseudohyphae and yeast aggregates.

Over time, treated surfaces tend to regain hydrophobicity, making them more prone to microbial colonization and reducing cell-surface interactions.^33^ Thus, performing treatment on samples still wrapped in surgical grade paper can prevent surface contamination with atmospheric hydrocarbons, also providing greater implant sterility assurance. Furthermore, lower hydrocarbon contamination is associated with a higher implant stability quotient, greater bone-implant contact and increased cell fixation, proliferation and differentiation.^33,34^ Therefore, the risk of contamination during pre- and intrasurgery is reduced^4^ and the consequent need for long-term preventive antimicrobial treatments, mitigating microbial resistance, in addition to hospitalizations and surgical interventions to correct implantation failures resulting from infections, reducing overall health care costs. Treatment immediately before implantation reduces the loss of treatment effects due to time,^4,35^ especially when in sealed packaging,^6,36^ ensuring greater orthopedic and dentistry therapy effectiveness.

Previous studies have demonstrated that this approach to implant surface modulation does not induce hemotoxicity on titanium surfaces under similar treatment conditions.^6^ This finding is particularly relevant, as the blood is the first tissue to interact with the implant surface, playing a critical role in forming the initial clot, which promotes osteoinduction and tissue integration.^37^ Additionally, our prior work^6^ demonstrated that the same treatment effectively modulates surface characteristics to inhibit bacterial adhesion, further supporting its potential for biomedical applications. However, despite these promising results, the present study has limitations. While this work focused on fungal contamination and surface modulation, further investigations are needed to evaluate the *in vivo* performance of treated implants, particularly in contact with osteoprogenitor cells, which are directly involved in osseointegration. Such studies would provide a more comprehensive understanding of the biocompatibility and clinical applicability of this approach to Ti-6Al-4 V alloys.

## Conclusions

The treatment of Ti-6Al-4 V alloy samples, previously sterilized and packaged in surgical-grade paper, using a cold atmospheric plasma (CAP) jet generated by a dielectric barrier discharge in a portable device, demonstrated significant potential for surface modulation. This innovative approach effectively altered the chemical composition of the biomaterial surface, enhancing hydrophilicity and significantly reducing the adhesion capacity of *Candida albicans*, as well as the formation of yeast aggregates and pseudohyphae.

These findings indicate that CAP treatment can serve as a complementary method to improve the performance of biomedical implants by potentially reducing the need for expensive antimicrobial therapies and mitigating the limitations of conventional sterilization methods. However, further studies, including *in vivo* evaluations and assessments of biocompatibility with osteoprogenitor cells, are required to validate the clinical applicability of this treatment and ensure its safety for broader use in Ti-6Al-4 V alloy-based devices.

## References

[ref1] Ghasemi-Mobarakeh L.; Kolahreez D.; Ramakrishna S.; Williams D. Key Terminology in Biomaterials and Biocompatibility. Curr. Opin Biomed Eng. 2019, 10, 45–50. 10.1016/j.cobme.2019.02.004.

[ref2] SARTORETTO S. C.; ALVES A. T. N. N.; RESENDE R. F. B.; CALASANS-MAIA J.; GRANJEIRO J. M.; CALASANS-MAIA M. D. Early Osseointegration Driven by the Surface Chemistry and Wettability of Dental Implants. Journal of Applied Oral Science 2015, 23 (3), 279–287. 10.1590/1678-775720140483. 26221922 PMC4510662

[ref3] Todros S.; Todesco M.; Bagno A. Biomaterials and Their Biomedical Applications: From Replacement to Regeneration. Processes 2021, 9 (11), 1949 10.3390/pr9111949.

[ref4] Berger M. B.; Cohen D. J.; Levit M. M.; Puetzer J. L.; Boyan B. D.; Schwartz Z. Hydrophilic Implants Generated Using a Low-Cost Dielectric Barrier Discharge Plasma Device at the Time of Placement Exhibit Increased Osseointegration in an Animal Pre-Clinical Study: An Effect That Is Sex-Dependent. Dental Materials 2022, 38 (4), 632–645. 10.1016/j.dental.2022.02.002. 35184898 PMC9123943

[ref5] Huo D.; Wang F.; Yang F.; Lin T.; Zhong Q.; Deng S.-P.; Zhang J.; Tan S.; Huang L. Medical Titanium Surface-Modified Coatings with Antibacterial and Anti-Adhesive Properties for the Prevention of Implant-Associated Infections. J. Mater. Sci. Technol. 2024, 179, 208–223. 10.1016/j.jmst.2023.09.016.

[ref6] Martins G. M.; da Silva Braz J. K. F.; de Macedo M. F.; de Oliveira Vitoriano J.; Alves Júnior C.; Santos C. S.; Feijó F. M. C.; de Oliveira M. F.; de Moura C. E. B. Enhancing Titanium Disk Performance through In-Pack Cold Atmospheric Plasma Treatment. ACS Biomater Sci. Eng. 2024, 10 (3), 1765–1773. 10.1021/acsbiomaterials.3c01388. 38357873

[ref7] Fan F.; Liu Y.; Liu Y.; Lv R.; Sun W.; Ding W.; Cai Y.; Li W.; Liu X.; Qu W. Candida Albicans Biofilms: Antifungal Resistance, Immune Evasion, and Emerging Therapeutic Strategies. Int. J. Antimicrob. Agents 2022, 60 (5–6), 106673 10.1016/j.ijantimicag.2022.106673. 36103915

[ref8] Ponde N. O.; Lortal L.; Ramage G.; Naglik J. R.; Richardson J. P. *Candida Albicans* Biofilms and Polymicrobial Interactions. Crit Rev. Microbiol 2021, 47 (1), 91–111. 10.1080/1040841X.2020.1843400. 33482069 PMC7903066

[ref9] Ramage G.; Martínez J. P.; López-Ribot J. L. *Candida* Biofilms on Implanted Biomaterials: A Clinically Significant Problem. FEMS Yeast Res. 2006, 6 (7), 979–986. 10.1111/j.1567-1364.2006.00117.x. 17042747

[ref10] Bongomin F.; Gago S.; Oladele R.; Denning D. Global and Multi-National Prevalence of Fungal Diseases—Estimate Precision. Journal of Fungi 2017, 3 (4), 57 10.3390/jof3040057. 29371573 PMC5753159

[ref11] Bürgers R.; Hahnel S.; Reichert T. E.; Rosentritt M.; Behr M.; Gerlach T.; Handel G.; Gosau M. Adhesion of Candida Albicans to Various Dental Implant Surfaces and the Influence of Salivary Pellicle Proteins. Acta Biomater 2010, 6 (6), 2307–2313. 10.1016/j.actbio.2009.11.003. 19925892

[ref12] Souza J. G. S.; Costa R. C.; Sampaio A. A.; Abdo V. L.; Nagay B. E.; Castro N.; Retamal-Valdes B.; Shibli J. A.; Feres M.; Barão V. A. R.; Bertolini M. Cross-Kingdom Microbial Interactions in Dental Implant-Related Infections: Is Candida Albicans a New Villain?. iScience 2022, 25 (4), 103994 10.1016/j.isci.2022.103994. 35313695 PMC8933675

[ref13] Tseng K.-Y.; Huang Y.-T.; Huang Y.-T.; Su Y.-T.; Wang A.-N.; Weng W.-Y.; Ke C.-L.; Yeh Y.-C.; Wang J.-J.; Du S.-H.; Gu Z.-Q.; Chen W.-L.; Lin C.-H.; Tsai Y.-H. Regulation of Candidalysin Underlies Candida Albicans Persistence in Intravascular Catheters by Modulating NETosis. PLoS Pathog 2024, 20 (6), e1012319 10.1371/journal.ppat.1012319. 38885290 PMC11213320

[ref14] Silva-Dias A.; Miranda I. M.; Branco J.; Monteiro-Soares M.; Pina-Vaz C.; Rodrigues A. G. Adhesion, Biofilm Formation, Cell Surface Hydrophobicity, and Antifungal Planktonic Susceptibility: Relationship among Candida Spp. Front Microbiol 2015, 6, 205 10.3389/fmicb.2015.00205. 25814989 PMC4357307

[ref15] Rutala W. A.; Weber D. J. Selection of the Ideal Disinfectant. Infect Control Hosp Epidemiol 2014, 35 (7), 855–865. 10.1086/676877. 24915214

[ref16] Weber D. J.; Rutala W. A. Use of Germicides in the Home and the Healthcare Setting Is There a Relationship Between Germicide Use and Antibiotic Resistance?. Infect Control Hosp Epidemiol 2006, 27 (10), 1107–1119. 10.1086/507964. 17006819

[ref17] Baumstark-Khan C.; Horneck G. Radiation Biology. In Encyclopedia of Astrobiology; Springer: Berlin Heidelberg: Berlin, Heidelberg, 2014; pp 1–3. 10.1007/978-3-642-27833-4_1332-3.

[ref18] Rutala W. A.; Weber D. J. Disinfection, Sterilization, and Control of Hospital Waste. In Mandell, Douglas, and Bennett’s Principles and Practice of Infectious Diseases; Elsevier, 2015; pp 3294–3309. 10.1016/B978-1-4557-4801-3.00301-5.

[ref19] Dhaliwal J. S.; David S. R. N.; Zulhilmi N. R.; Sodhi Dhaliwal S. K.; Knights J.; de Albuquerque Junior R. F. Contamination of Titanium Dental Implants: A Narrative Review. SN Appl. Sci. 2020, 2 (6), 1011 10.1007/s42452-020-2810-4.

[ref20] Donos N.; Calciolari E. Dental Implants in Patients Affected by Systemic Diseases. Br Dent J. 2014, 217 (8), 425–430. 10.1038/sj.bdj.2014.911. 25342349

[ref21] Rupf S.; Lehmann A.; Hannig M.; Schäfer B.; Schubert A.; Feldmann U.; Schindler A. Killing of Adherent Oral Microbes by a Non-Thermal Atmospheric Plasma Jet. J. Med. Microbiol 2010, 59 (2), 206–212. 10.1099/jmm.0.013714-0. 19910483

[ref22] Graves D. B. The Emerging Role of Reactive Oxygen and Nitrogen Species in Redox Biology and Some Implications for Plasma Applications to Medicine and Biology. J. Phys. D Appl. Phys. 2012, 45 (26), 263001 10.1088/0022-3727/45/26/263001.

[ref23] Sharma S.; Jaiswal S.; Duffy B.; Jaiswal A. K. Advances in Emerging Technologies for the Decontamination of the Food Contact Surfaces. Food Research International 2022, 151, 110865 10.1016/j.foodres.2021.110865. 34980401

[ref24] Maria A.; De Melo C.; Myrza T.; Cardoso S.; Rocha V.; Cruz A.; Alessandra C.; De Oliveira A.; Vital C.; Pastl R. M.; Carvalho S.; Carcinoma de Células Escamosas Em Felino: Relato de Caso Squamous Cells Carcinoma in Feline; Pubvet 2018, 1–6.

[ref25] Alves Junior C.; de Oliveira Vitoriano J.; da Silva D. L. S.; de Lima Farias M.; de Lima Dantas N. B. Water Uptake Mechanism and Germination of Erythrina Velutina Seeds Treated with Atmospheric Plasma. Sci. Rep 2016, 6 (1), 33722 10.1038/srep33722. 27670654 PMC5037400

[ref26] Braz J. K. F. S.; Martins G. M.; Sabino V.; Vitoriano J. O.; Barboza C. A. G.; Soares A. K. M. C.; Rocha H. A. O.; Oliveira M. F.; Alves Júnior C.; Moura C. E. B. Plasma Nitriding under Low Temperature Improves the Endothelial Cell Biocompatibility of 316L Stainless Steel. Biotechnol. Lett. 2019, 41 (4–5), 503–510. 10.1007/s10529-019-02657-7. 30820710

[ref27] Ma C.; Nikiforov A.; Hegemann D.; De Geyter N.; Morent R.; Ostrikov K. Plasma-Controlled Surface Wettability: Recent Advances and Future Applications. Int. Mater. Rev. 2023, 68 (1), 82–119. 10.1080/09506608.2022.2047420.

[ref28] Wei G.; Tan M.; Attarilar S.; Li J.; Uglov V. V.; Wang B.; Liu J.; Lu L.; Wang L. An Overview of Surface Modification, A Way toward Fabrication of Nascent Biomedical Ti–6Al–4V Alloys. Journal of Materials Research and Technology 2023, 24, 5896–5921. 10.1016/j.jmrt.2023.04.046.

[ref29] Melentiev R.; Fang F.; Narala S. K. R. Influence of Different Pretreatments on Ti-6Al-4V Surface Integrity and Scratch-Resistance of Epoxy Coating: Analysis of Topography, Microstructure, Chemistry and Wettability. Surf. Coat. Technol. 2020, 404, 126436 10.1016/j.surfcoat.2020.126436.

[ref30] Alves Junior C. Plasma Frio Atmosférico – Novas Oportunidades de Pesquisa Numa Plataforma Versátil e Portadora de Futuro. Matéria 2020, 25 (4), e-12912 10.1590/s1517-707620200004.1212.

[ref31] Choi E. H.; Uhm H. S.; Kaushik N. K. Plasma Bioscience and Its Application to Medicine. AAPPS Bulletin 2021, 31 (1), 10 10.1007/s43673-021-00012-5.

[ref32] Li J.; Hirota K.; Goto T.; Yumoto H.; Miyake Y.; Ichikawa T. Biofilm Formation of Candida Albicans on Implant Overdenture Materials and Its Removal. J. Dent 2012, 40 (8), 686–692. 10.1016/j.jdent.2012.04.026. 22580351

[ref33] Naauman Z.; Rajion Z. A. B.; Maliha S.; Hariy P.; Muhammad Q. S.; Noor H. A. R. Ultraviolet A and Ultraviolet C Light-Induced Reduction of Surface Hydrocarbons on Titanium Implants. Eur. J. Dent 2019, 13 (01), 114–118. 10.1055/s-0039-1688741. 31170762 PMC6635973

[ref34] Arroyo-Lamas N.; Arteagoitia I.; Ugalde U. Surface Activation of Titanium Dental Implants by Using UVC-LED Irradiation. Int. J. Mol. Sci. 2021, 22 (5), 2597 10.3390/ijms22052597. 33807532 PMC7961349

[ref35] Chen F.; Xu W.; Huang S.; Liu J.; Song J.; Liu X. Plasma Hydrophilization of Superhydrophobic Surface and Its Aging Behavior: The Effect of Micro/Nanostructured Surface. Surf. Interface Anal. 2016, 48 (6), 368–372. 10.1002/sia.5988.

[ref36] Benčina M.; Resnik M.; Starič P.; Junkar I. Use of Plasma Technologies for Antibacterial Surface Properties of Metals. Molecules 2021, 26 (5), 1418 10.3390/molecules26051418. 33808010 PMC7961478

[ref37] Shiu H. T.; Goss B.; Lutton C.; Crawford R.; Xiao Y. Formation of Blood Clot on Biomaterial Implants Influences Bone Healing. Tissue Eng. Part B Rev. 2014, 20 (6), 697–712. 10.1089/ten.teb.2013.0709. 24906469

